
JOURNAL INFORMATION
==============================
NLM Title Abbreviation: Behav Neurol
Journal ID: Behav Neurol
NLM Journal ID: 8914585
Journal ID: BN

Behavioural Neurology

ISSN: 0953-4180
EISSN: 1875-8584
Publisher: Wiley

ARTICLE INFORMATION
==============================
PMCID: PMC5434410
PMID: 21422565
DOI: 10.3233/BEN-2010-0305
Article ID: 572174
Article version: 1
Subjects: Research Article

The Role of Reality Monitoring in Anosognosia for Hemiplegia

Jenkinson Paul M. 1 *
Edelstyn Nicola M. J. 2 3
Drakeford Justine L. 4
Roffe Christine 3
Ellis Simon J. 5
1 School of Psychology University of Hertfordshire Hatfield UK
2 School of Psychology Keele University Keele UK
3 Research Institute for Life Course Studies Keele University Keele UK
4 School of Human and Health Sciences University of Huddersfield Huddersfield UK
5 Department of Neurology University Hospital of North Staffordshire Stoke-On-Trent UK
*Paul M. Jenkinson: dr.paul.jenkinson@gmail.com
Print publication date: 2010
Electronic publication date: 2011 Mar 7
Volume: 23
Issue: 4
First page: 241
Last page: 243
Received 2011 Jan 31; Accepted 2011 Jan 31
Copyright: Copyright © 2010 Hindawi Publishing Corporation and the authors.
Copyright year: 2010
License: This is an open access article distributed under the Creative Commons Attribution License, which permits unrestricted use, distribution, and reproduction in any medium, provided the original work is properly cited.
License URL: https://creativecommons.org/licenses/by/3.0/

==============================
Behavioural Neurology 23 (2010) 241–243
DOI 10.3233/BEN-2010-0305
IOS Press

### The role of reality monitoring in anosognosia for hemiplegia

Paul M. Jenkinson[a,*], Nicola M. J. Edelstyn[b,c], Justine L. Drakeford[d], Christine Roffe[c] and Simon J. Ellis[e]
[a]*School of Psychology, University of Hertfordshire, Hatfield, UK*
[b]*School of Psychology, Keele University, Keele, UK*
[c]*Research Institute for Life Course Studies, Keele University, Keele, UK*
[d]*School of Human and Health Sciences, University of Huddersfield, Huddersfield, UK*
[e]*Department of Neurology, University Hospital of North Staffordshire, Stoke-On-Trent, UK*

### 1. Introduction

Anosognosia for hemiplegia (AHP) refers to a lack of awareness regarding paralysis after stroke. Despite attracting clinical interest for decades, empirical research into AHP has been relatively scarce, and there remains no universally accepted explanation [5]. This is partially due to difficulty characterising the disorder. The term has been applied to both partial and complete lack of awareness, with partial unawareness presenting as a failure to recognise, appreciate the severity, or acknowledge the consequences of paralysis, and more complete cases involving a failure to admit the presence of a paralysis even after its demonstration [8]. The fact that some patients verbally deny their problems, but show behaviours consistent with their paralysis (e.g. executing a bi-manual tasks using a unimanual strategy), while others verbally accept their paralysis but behave in a manner inconsistent with this acceptance (e.g. attempting to walk), suggests that verbal and behavioural awareness are independent [4]. The observation of diverse lesion sites, emotional, perceptual, and cognitive impairments in anosognosia has also resulted in unawareness being considered a multifaceted or multicomponent disorder involving several subtypes [4, 8,10]. As such, different forms of anosognosia may reflect the combination of various deficits, the exact components of which are not currently known [10].

Recent accounts of AHP have employed a model of the motor system, which proposes that awareness involves a comparison of predicted and actual sensory information (Fig. 1). It is suggested that AHP patients fail to register discrepancy between internal sensory predictions and external sensory information [1, 3]. This results in an erroneous feeling of having performed intended movements using the paralysed limb. Recent experiments in AHP support this idea; however, a purely motor account cannot explain several aspects of AHP, such as its delusional character (e.g. resilience to counterargument) and associated affective disturbance [5]. It is likely that other neurocognitive disturbances also contribute to AHP. On the basis of research in other delusional patients [2], and a speculated reality monitoring impairment in an AHP case-report [9], we hypothesised that an inability to discriminate between internally- and externally-generated information (i.e. reality monitor) would contribute to AHP.

We conducted two experiments to examine this proposal [6]. Experiment 1 employed a classic reality monitoring paradigm [7], to test the ability to discriminate between seen (perceived) and imagined drawings of objects in AHP patients ($n = 10$), hemiplegic con-

*Corresponding author: Dr. Paul Jenkinson, School of Psychologym, University of Hertfordshire, Hatfield, AL10 9AB, UK. E-mail: dr.paul.jenkinson@gmail.com.

ISSN 0953-4180/10/$27.50 © 2010 – IOS Press and the authors. All rights reserved

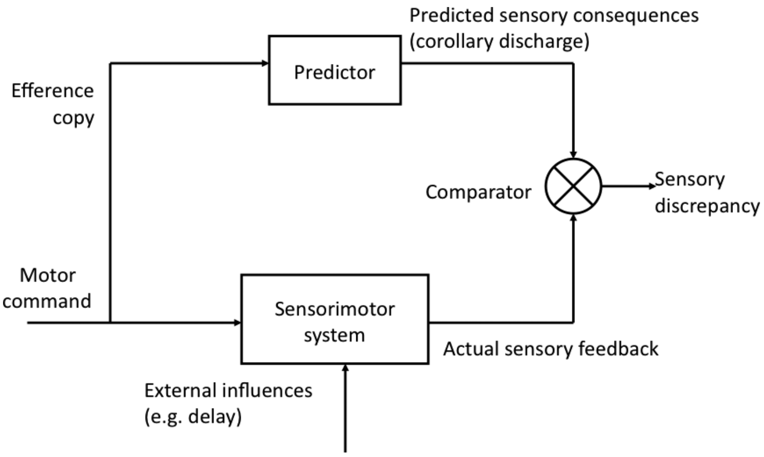

Fig. 1. A simple model of the motor system adapted from Frith et al. [3]. Actual and predicted sensory information are compared to generate a sensory discrepancy which signals movement error.

trol patients without AHP (nonAHP, $n = 7$) and age-matched healthy controls (HC, $n = 20$). During a study phase, subjects were presented with a word (e.g. PEN) followed by either: (i) a picture representing the object (i.e. drawing of a pen), or (ii) an empty circle into which they projected a mental picture of the previous word (i.e. imagine a drawing of a pen). In a test phase, subjects saw previously studied (target) and unstudied (new) words, and had to decide if each word had been studied previously. Following this, words identified as previously studied were assessed in terms of the source of the original image (i.e. "Did you previously *see* or *imagine* a drawing of a [PEN]?"). AHP patients were significantly impaired at this task relative to HCs (omnibus Kruskal-Wallis test $H(2)= 21.23, p < 0.001$) and nonAHP patients (post-hoc Mann-Whitney $U = 6, p = 0.006$), indicating a deficit in discriminating the source of images as real/imagined.

A second experiment explored if this reality monitoring deficit occurs in the motor domain. Adapting the procedure of Experiment 1, AHP ($n = 3$), nonAHP ($n = 6$), and HC ($n = 20$) subjects were presented orally with action phrases (e.g. point to the door), which they either had to execute themselves (perform item), imagine executing (imagine item), or observe the experimenter executing (observe item). The ability to discriminate studied/unstudied phrases, and make source judgements was then assessed using the procedure of Experiment 1 (e.g. "Did you previously [perform/imagine/observe] pointing to the door?"). Results again indicated impaired reality monitoring in patients with AHP compared with HCs ($H(2) = 11.54$, $p = 0.001$) and nonAHP patients ($U = 1, p = 0.048$). However, the ability to reality monitor movements was also impaired in nonAHP patients relative to HCs ($U = 24$, $p = 0.026$). Performance on the task showed a steady decline from HC levels, to mild impairment in nonAHP patients, and greatest impairment in AHP.

Findings of these two experiments suggest a combination of reality monitoring impairments in the pathogenesis of AHP. Experiment 1 showed that the ability to discriminate between real and imagined drawings was impaired in AHP patients only. Experiment 2 showed that the ability to monitor actions is deficient in both AHP and nonAHP patients, but is more impaired in patients with AHP. As such, impaired reality monitoring of movement might be a general consequence of damage to the motor system. From our results it is not possible to identify whether the processes responsible for greater impairment of action reality monitoring in AHP are the same as those underlying the deficit observed in nonAHP patients.

We speculate that this combination of reality monitoring deficits prevents AHP patients from checking the veracity of knowledge about the motor system and their current state generally. This is consistent with the ABC model [10], in which awareness relies on an ability to Check available information, in order to change one's Beliefs, and/or act upon signals of uncertainty arising from subjective Appreciation (experience) of a specific function (e.g. moving). This explanation readily accommodates recent motor explanations of AHP [1,3]: the erroneous belief that one is able to move may arise from a defective appreciation of paralysis, caused by impaired sensory feedback, and/or a failure to register discrepancies between motor intentions and sensory information. This explanation suggests that AHP can result from different deficits which combine to produce the same clinical endpoint. As such, it is able to account for the multifaceted nature of AHP; however, further research is needed to identify the exact factors which produce AHP.

### References

[1] A. Berti, L. Spinazzola, L. Pia, M. Rabuffetti, P. Haggard, Y. Rossetti et al., Motor awareness and motor intention in anosognosia for hemiplegia, in: *Sensorimotor foundations of higher cognition*, Oxford: Oxford University Press, 2007, pp. 163–181.

[2] G. Brébion, M. Smith, J. Gorman and X. Amador, Discrimination accuracy and decision biases in different types of reality monitoring in schizophrenia. *Journal of Nervous and Mental Disease* **185** (1997), 247–253.

[3] C.D. Frith, S.J. Blakemore and D.M. Wolpert, Abnormalities in the awareness and control of action. *Philosophical Transactions of the Royal Society of London: Biological Sciences* **355** (2000), 1771–1788.

[4] M. Jehkonen, M. Laihosalo and J. Kettunen, Anosognosia after stroke: Assessment, occurrence, subtypes and impact on functional outcome reviewed, *Acta Neurologica Scandinavica* **114** (2006), 293–306.

[5] P.M. Jenkinson and A. Fotopoulou, Motor awareness in anosognosia for hemiplegia: Experiments at last! *Experimental Brain Research* **204** (2010), 295–304.

[6] P.M. Jenkinson, N.M.J. Edelstyn, J.L. Drakeford and S.J. Ellis, Reality monitoring in anosognosia for hemiplegia, *Consciousness and Cognition* **18** (2009), 458–470.

[7] M.K. Johnson, Reality monitoring: Evidence from confabulation in organic brain disease patients, in: *Awareness of deficit after brain injury: Clinical and theoretical issues*, G.P. Prigatano and D.L. Schacter, eds, New York: Oxford University Press, 1991, pp. 176–197.

[8] M.D. Orfei, R.G. Robinson, G.P. Prigatano, S. Starkstein, N. Rüsch, P. Bria et al., Anosognosia for hemiplegia after stroke is a multifaceted phenomenon: A systematic review of the literature, *Brain* **130**(12) (2007), 3075–3090.

[9] A. Venneri and M.F. Shanks, Belief and awareness: reflections on a case of persistent anosognosia, *Neuropsychologia* **42** (2004), 230–238.

[10] R. Vocat and P. Vuilleumier, Neuroanatomy of impaired body awareness in anosognosia and hysteria: A multicomponent account, in: *The study of anosognosia*, G. Prigatano, ed., New York, USA: Oxford University Press, 2010, pp. 359–403.
